# Dynamics of peripheral blood inflammatory index predict tumor pathological response and survival among patients with locally advanced non-small cell lung cancer who underwent neoadjuvant immunochemotherapy: a multi-cohort retrospective study

**DOI:** 10.3389/fimmu.2024.1422717

**Published:** 2024-07-23

**Authors:** Wenyu Zhai, Chao Zhang, Fangfang Duan, Jingdun Xie, Shuqin Dai, Yaobin Lin, Qihang Yan, Bingyu Rao, Liang Li, Yuheng Zhou, Zerui Zhao, Hao Long, Junye Wang

**Affiliations:** ^1^ Department of Thoracic Surgery, State Key Laboratory of Oncology in Southern China, Collaborative Innovation Center for Cancer Medicine, Guangdong Provincial Clinical Research Center for Cancer, Sun Yat-Sen University Cancer Center, Guangzhou, China; ^2^ Lung Cancer Research Center, Sun Yat-Sen University, Guangzhou, China; ^3^ Department of Breast Oncology, State Key Laboratory of Oncology in Southern China, Collaborative Innovation Center for Cancer Medicine, Guangdong Provincial Clinical Research Center for Cancer, Sun Yat-Sen University Cancer Center, Guangzhou, China; ^4^ Department of Anesthesiology, State Key Laboratory of Oncology in Southern China, Collaborative Innovation Center for Cancer Medicine, Guangdong Provincial Clinical Research Center for Cancer, Sun Yat-Sen University Cancer Center, Guangzhou, China; ^5^ Department of Medicine Laboratory, State Key Laboratory of Oncology in Southern China, Collaborative Innovation Center for Cancer Medicine, Guangdong Provincial Clinical Research Center for Cancer, Sun Yat-Sen University Cancer Center, Guangzhou, China

**Keywords:** non-small cell lung cancer, neoadjuvant immunotherapy, tumor biomarkers, longitudinal dynamics, transcriptomic analysis

## Abstract

**Background:**

Static tumor features before initiating anti-tumor treatment were insufficient to distinguish responding from non-responding tumors under the selective pressure of immuno-therapy. Herein we investigated the longitudinal dynamics of peripheral blood inflammatory indexes (dPBI) and its value in predicting major pathological response (MPR) in non-small cell lung cancer (NSCLC).

**Methods:**

A total of 147 patients with NSCLC who underwent neoadjuvant immunochemotherapy were retrospectively reviewed as training cohort, and 26 NSCLC patients from a phase II trial were included as validation cohort. Peripheral blood inflammatory indexes were collected at baseline and as posttreatment status; their dynamics were calculated as their posttreatment values minus their baseline level. Least absolute shrinkage and selection operator algorithm was utilized to screen out predictors for MPR, and a MPR score was integrated. We constructed a model incorporating this MPR score and clinical predictors for predicting MPR and evaluated its predictive capacity via the area under the curve (AUC) of the receiver operating characteristic and calibration curves. Furthermore, we sought to interpret this MPR score in the context of micro-RNA transcriptomic analysis in plasma exosomes for 12 paired samples (baseline and posttreatment) obtained from the training cohort.

**Results:**

Longitudinal dynamics of monocyte–lymphocyte ratio, platelet-to-lymphocyte ratio, platelet-to-albumin ratio, and prognostic nutritional index were screened out as significant indicators for MPR and a MPR score was integrated, which was further identified as an independent predictor of MPR. Then, we constructed a predictive model incorporating MPR score, histology, and differentiated degree, which discriminated MPR and non-MPR patients well in both the training and validation cohorts with an AUC value of 0.803 and 0.817, respectively. Furthermore, micro-RNA transcriptomic analysis revealed the association between our MPR score and immune regulation pathways. A significantly better event-free survival was seen in subpopulations with a high MPR score.

**Conclusion:**

Our findings suggested that dPBI reflected responses to neoadjuvant immuno-chemotherapy for NSCLC. The MPR score, a non-invasive biomarker integrating their dynamics, captured the miRNA transcriptomic pattern in the tumor microenvironment and distinguished MPR from non-MPR for neoadjuvant immunochemotherapy, which could support the clinical decisions on the utilization of immune checkpoint inhibitor-based treatments in NSCLC patients.

## 1 Introduction

Immune checkpoint inhibitors (ICIs) targeting the interaction of programmed cell death protein-1 (PD-1) with its ligand PD-L1 as a single agent or plus other anti-tumor therapies have dramatically revolutionized the management of non-small cell lung cancer (NSCLC) and significantly improved patients’ clinical outcomes (1). Therefore, ICIs are moving forward to the neoadjuvant setting (2–4) and have been approved by the US Food and Drug Administration as a neoadjuvant treatment for patients with locally advanced NSCLC (5). While nearly half of NSCLC patients failed to respond to ICI-based neoadjuvant therapy, a proportion of them even undergo hyper-progress. Moreover, immune-related toxicity might hinder subsequent surgery and be even fatal (6). Hence, precise and reliable approaches to predict therapeutic efficacy and identify ideal responders to ICI-based neoadjuvant treatment are of great importance.

Current predominantly utilized biomarkers in clinic and trials design are PD-L1 expression and tumor mutation burden (TMB), although considerable efforts have been made to develop them as companion biomarkers. Most NSCLC patients with high PD-L1 expression and TMB show no long-term benefits from ICIs, while some patients with low PD-L1 and TMB tumors are responders (7, 8). Both PD-L1 and TMB heavily depend on tissues and are subject to technical challenges and clinical specimens. Dynamic reflection of responses to neoadjuvant immunochemotherapy is difficult to be reflected. In addition, accurate estimates of PD-L1 and TMB might be impacted by tumor heterogeneity and purity, making them insufficient to accurately predict outcomes to ICIs (9, 10). Latest research demonstrated that genetic phenotype rather than mutation status was crucial for responders’ selection (11). Other potential predictors, such as tumor-infiltrating lymphocytes, microbiome, multi-omics, and so on (12), are limited by high cost, time-consuming for operation, and required tissue specimens. Thus, exploiting economic and reliable biomarkers to identify NSCLC patients responding to neoadjuvant immunochemotherapy is urgently required and meaningful.

In recent years, increasing focus on peripheral blood inflammation indexes, such as hemoglobin, lactate dehydrogenase, neutrophil-to-lymphocyte ratio (NLR), platelet-to-lymphocyte ratio (PLR), monocyte–lymphocyte ratio (MLR), and platelet-to-albumin ratio (PAR), which represent systemic immune-inflammatory status and tumor burden, has been made to predict the therapeutic efficacy of NSCLC patients. Their lower baseline level or post-treatment reduction was widely considered to be associated with the higher anti-tumor response rate and better long-term outcomes in patients (6, 13–16). However, most prior studies were either based on monotherapy or just paid attention to the pretreatment baseline level without clear evidence of their dynamics during immunotherapy. Importantly, specific inflammation alterations could shape the tumor microenvironment (TME), and response to anti-tumor immune is orchestrated by immune-related pathways. The complex crosstalk between tumor and immune cells during ICI-based treatment highlights the need to develop integrated models to interpret immunotherapy responses and predict clinical outcomes, while static single feature analyses are insufficient to capture the dynamic nature and plasticity of the tumor–immune system interplay during immune checkpoint blockade (14, 17). Hence, tracking the longitudinal dynamics of peripheral blood inflammatory indexes (dPBI) during anti-tumor immune treatment gradually increased the attention and interest of researchers.

Herein we conducted an integrative analysis of dPBI during neoadjuvant immuno-chemotherapy to predict the therapeutic responses for NSCLC patients with the help of the incorporated multi-retrospective cohorts. Ultimately, we modeled their dynamics using a score and linked this score with therapeutic responses at the cellular level (evaluated by major pathological response, MPR) and the molecular level [assessed by micro-RNA (miRNA) transcriptomic analysis of plasma exosomes].

## 2 Materials and methods

### 2.1 Study design and patient population

**Figure 1:**
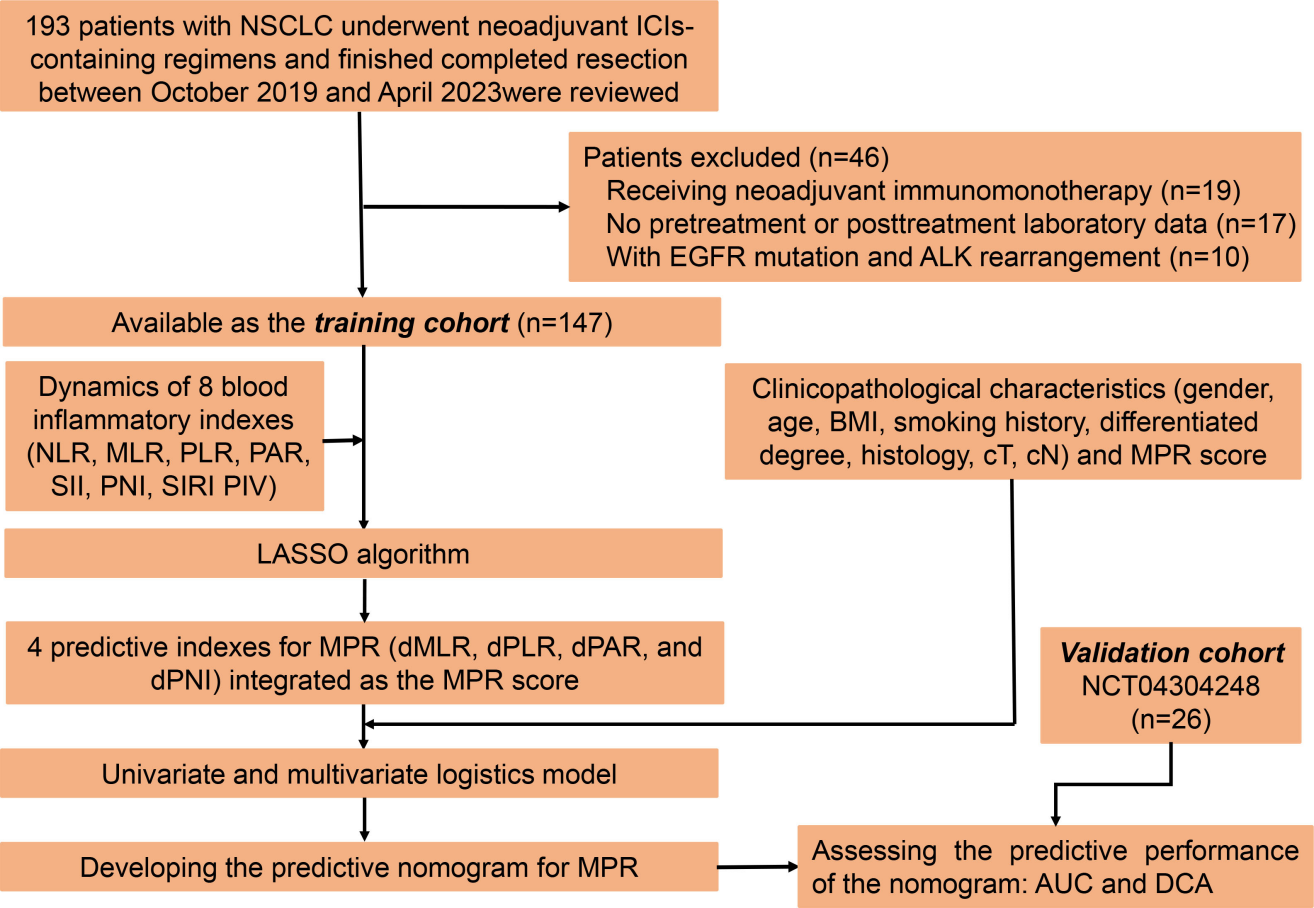
Study enrollment, source and distribution of cases, and identification of predictive indexes for major pathological response.

This is a multi-cohort retrospective study. For the training cohort, we retrospectively reviewed the medical records of 193 NSCLC patients who underwent neoadjuvant ICI-containing regimens and had completed resection between October 2019 and April 2023 at our center. After rigorous screening, 147 patients were included in the study (**Figure 1**). The key inclusion criteria were as follows: (1) above 18 years old, (2) pathologically confirmed as NSCLC, (3) clinically staged IIA–IIIB (cT1–4N0–3) according to the eighth edition of the American Joint Committee on Cancer stage system, (4) administration of at least two cycles of neoadjuvant immunotherapy, (5) resection specimens subjected to pathological assessment after neoadjuvant immunochemotherapy, and (6) complete follow-up and clinicopathological information. Patients meeting the following exclusion criteria were ineligible: (1) receiving neoadjuvant immunomonotherapy, (2) with epidermal growth factor receptor (EGFR) gene mutation and anaplastic lymphoma kinase (ALK) gene rearrangement, (3) lacking peripheral blood laboratory data within 1 week before neoadjuvant immunochemotherapy, (4) a history of malignancies at other sites, and (5) suffering from active acute infection or chronic infection or steroid treatments within 1 month before the neoadjuvant immunochemotherapy. As for the validation cohort, patients with NSCLC from our previously published prospective, phase II study (NCT04304248) were analyzed (18).

This study was performed following the recommendations of the Declaration of Helsinki, and its protocol was reviewed and approved by the ethics committee (no. B2022–445-01). The requirement for written informed consent from patients was waived owing to its retrospective nature.

### 2.2 Definition of peripheral blood inflammatory indexes and data collection

Peripheral blood values were manually retrieved from the medical records at baseline (within 1 week before the first neoadjuvant treatment) and posttreatment (within 1 week before resection). Specifically, we collected the counts of neutrophils (10^9^/L, N), monocytes (10^9^/L, M), platelets (10^9^/L, P), lymphocytes (10^9^/L, L), and serum albumin concentrations (10^5^/L, A). The specific peripheral blood inflammatory indexes were calculated as follows: NLR = N/L (14), MLR = M/L (19), PLR = P/L (20), platelet–albumin ratio (PAR) = P/A (21), prognostic nutritional index (PNI) = A+5*L (22), systemic immune inflammation index (SII) = P*NLR (23), systemic inflammation response index (SIRI) = N*MLR (24), and PIV = N*M*P/L (13). Subsequently, the dynamic changes of these peripheral blood inflammatory indexes, which were named as dNLR, dMLR, dPLR, dPAR, dPNI, dSII, dSIRI, and dPIV, were defined as peripheral blood inflammatory indexes at the posttreatment point minus the corresponding value at baseline.

In addition, we collected data on clinicopathological factors of all patients included, including gender, age, body mass index (BMI) (obtained within 1 week before the first neoadjuvant treatment), smoking history, histological type, differentiation degree, cT stage, cN stage, and pathological response from the medical records.

### 2.3 Neoadjuvant therapy and pathological assessment

Neoadjuvant immunochemotherapy is administered every 3 weeks, and the ICI agent included PD-1 and PD-L1 inhibitors. Surgery is performed 4 to 6 weeks after the end of the last cycle of neoadjuvant immunochemotherapy.

Based on the multidisciplinary recommendations from the International Association for Lung Cancer Research (25) and published studies, MPR is defined as the presence of no more than 10% residual cancer cells within the primary tumor bed, which is the same as the Checkmate 159 and NADIM study (2, 26).

### 2.4 Follow-up of patients and study endpoints

We regularly monitored the patients’ medical conditions every 3 months through a telephone follow-up or by outpatient electronic records, including physical examinations, hematological and laboratory examinations, and chest and abdominal computed tomography (CT). Positron emission tomography CT, brain magnetic resonance imaging (MRI), and tracheoscopy were performed if necessary. In cases where the patients had passed away, the cause and date of death were also recorded during the follow-up process.

The main endpoint of this study is MPR. The secondary study endpoint is event-free survival (EFS), defined as the time interval from the date of initiation of neoadjuvant immunotherapy to the date of death or tumor recurrence.

### 2.5 Identifying a MPR signature based on dPBI

In the training cohort, we utilized the “glmnet” R package to perform least absolute shrinkage and selection operator (LASSO) algorithm to select the dPBI for predicting MPR and calculating their corresponding coefficients. On the basis of the results of the above-mentioned LASSO algorithm, a MPR signature was constructed, and its predictive score (MPR score) was summed up using the dynamic change of specific peripheral blood indexes and their corresponding regression coefficients. The specific formula was as follows: MPR score = sum (selected dPBI × corresponding coefficients).

### 2.6 Purification and identification of plasma exosome and exosomal miRNA sequencing

For miRNA sequencing, we collected plasma (within 1 week before the first neoadjuvant treatment) and posttreatment (within 1 week before resection) from 12 patients with NSCLC in the training cohort. Total exosome was extracted from 200 μL of plasma via the GS Reagent DF Kit (GENESEED, Guangzhou) following the manufacturer’s instructions. Then, we utilized the transmission electron microscope (HT-7700, Hitachi) to identify the purified exosomes, which were subsequently suspended in 100 μL of phosphate-buffered saline (PBS) and dropped on copper-coated grids. Before photographing by electron microscopy, the copper-coated grids were dried at room temperature after staining with 2% uranyl acetate. Approximately 100 ng of total RNA was used to prepare a small RNA library according to the protocol of TruSeq Small RNA Sample Prep Kits (Illumina, San Diego, CA, USA). Finally, we performed single-end sequencing (1 × 50 bp) on an Illumina Hiseq2500 at the LC-BIO (Hangzhou, China) following the vendor’s recommended protocol.

### 2.7 Processing of sequencing data and gene set enrichment analysis

Processing of raw data and miRNA mapping were achieved using the ACGT101-miR (LC Sciences, Houston, TX, USA) and miRBase 22.0 (http://www.mirbase.org/), and miRNA expression data were normalized for transcripts per kilobase of exon model per million mapped reads (TPM). The details of the above-mentioned procedure are provided in the **Supplementary Methods**.

We carried out the differential expression (DE) analysis via “limma” R package (27) to identify the DE miRNAs at baseline and after neoadjuvant immunochemotherapy. A miRNA with log2 | fold change | > 1 and *P*-value <0.05 were defined as DE miRNA, whose dynamic change was calculated as TPM at posttreatment – TPM at baseline. Then, Pearson correlation analysis was performed to recognize the correlation between the dynamic change of DE miRNA and MPR signature. Correlation coefficient (*R* ^2^) > 0.5 and *P*-value < 0.05 were considered as significantly correlated. We used two computational target prediction algorithms (TargetScan 5.0 and miRanda 3.3a) (28, 29) to predict genes targeted by miRNAs correlated with MPR signature. Gene with miranda Energy <-10 in miRanda algorithm and context score percentile >50 were identified as targeted genes of miRNAs. Subsequently, we executed the Kyoto Encyclopedia of Genes and Genomes (KEGG) enrichment analysis of targeted genes through the “clusterProfiler” R package (30), in which the pathway with *P*-value <0.05 was seen as apparent enrichment.

### 2.8 Statistical analysis

Continuous variables were shown as median values with interquartile ranges; for continuous variables with normal distribution, we used Student’s *t*-test to compare, and as for those not conforming to normal distribution, Mann–Whitney *U*-test was used to compare. Categorical variables were listed as count (percentage) and compared via the chi-squared test or Fisher’s exact test. The survival curves for EFS were estimated using the Kaplan–Meier method and compared through the log-rank test.

The univariate and multivariate logistic regression model was performed to identify the predictive value of MPR score and other independent predictors of MPR, based on which a predictive model for MPR was established and graphically presented as a nomogram through the “rms” R package. We further internally evaluated the predictive performance of this nomogram by calculating the area under the curve (AUC) of the receiver operating characteristic (ROC) and calibration curves. We performed 1,000 times bootstrap resampling to validate its predictive capability. Furthermore, we also externally evaluated the predictive performance of this nomogram via the AUC of ROC and calibration curves in the validation cohort. Ultimately, the clinical value of our nomogram was assessed by decision curve analysis (DCA), which could assess the net benefit of patients from this nomogram. The maximally selected log-rank test was used via the “maxstat” R package to determine the cutoff value of MPR score for converting the MPR score into a binary categorical variable.

Statistical analyses in this study were performed using R software (version 4.2.1, Vanderbilt University, Nashville, TN, USA) and SPSS software (version 22.0, SPSS Inc., Chicago, IL, USA). A two-tailed *P*-value <0.05 was considered statistically significant.

## 3 Results

### 3.1 Workflow of this study

**Figure 2:**
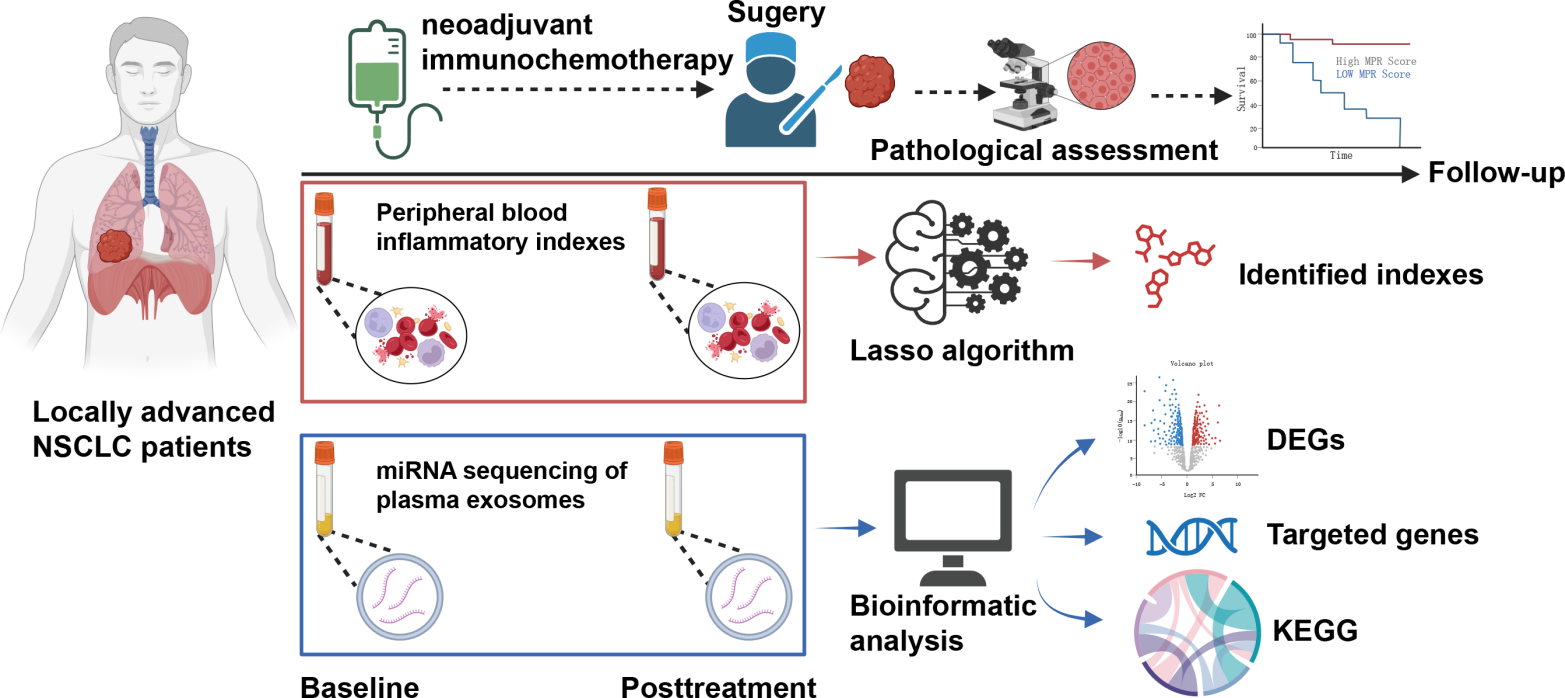
Flowchart for a comprehensive analysis of the dynamics of peripheral blood inflammatory index (dPBI) in patients with locally advanced non-small cell lung cancer who underwent neoadjuvant immunochemotherapy.

Herein a total of 147 patients were eligible for the training cohort. As for the validation cohort, 26 patients without EGFR mutation and ALK rearrangement from our previously published prospective, phase II study (NCT04304248) were enrolled (18). All patients enrolled here underwent ICIs plus chemotherapy as inductive treatment and then received surgery. Details of their neoadjuvant regimens are listed in **Supplementary Table S1**. The flow diagram of this work is shown in **Figure 2**.

### 3.2 Characteristics of the patients

The median age of the training cohort was 61 years old, while it was 59 years old in the validation cohort. Lung squamous carcinoma (LUSC) was the major histological type, accounting for 66.0% of the training cohort and 73.1% of the validation cohort. Male patients were significantly more than female patients in both the training cohort (89.1% *versus* 10.9%) and the validation cohort (84.6% *versus* 15.6%). Before neoadjuvant therapy, at least half of the patients were diagnosed as cN2 stage in both the training cohort (50.3%) and the validation cohort (50.0%). After neoadjuvant therapy, 101 (68.7%) and 20 (76.9%) patients in the training and validation cohorts achieved MPR, respectively. Moreover, 58 (39.5%) and 15 (57.7%) patients in the training and validation cohorts achieved pCR, respectively. Except for differentiation degree, the training cohort and the validation cohort were matched well. Compared with the validation cohort, more patients were moderately differentiated in the training cohort (35.4% *versus* 11.5%, *P* = 0.010) (**Table 1**).

**Table 1:** Patients’ characteristics.

Characteristics	Training cohort *N* = 147	Validation cohort *N* = 26	*P*-value
Gender			0.509
Male	131 (89.1)	22 (84.6)	
Female	16 (10.9)	4 (15.4)	
Age (year), median (IQR)	61.0 (56.0–66.0)	59.0 (55.8–66.3)	0.399
BMI	22.6 (20.5–25.2)	22.8 (21.4–25.0)	0.678
Smoking history			0.850
No	37 (25.2)	7 (26.9)	
Yes or ever	110 (74.8)	43 (73.1)	
cT stage^a^			0.156
T1	9 (6.1)	0 (0)	
T2	59 (40.1)	13 (50.0)	
T3	46 (31.3)	10 (38.5)	
T4	33 (22.4)	3 (11.5)	
cN stage^a^			0.563
N0	17 (11.6)	1 (3.8)	
N1	30 (20.4)	7 (26.9)	
N2	74 (50.3)	13 (50.0)	
N3	26 (17.7)	5 (19.2)	
Differentiation degree			**0.010**
Moderate	52 (35.4)	3 (11.5)	
Poor	89 (60.5)	19 (73.1)	
Undifferentiated	6 (4.1)	4 (15.4)	
Histological type			0.565
LUSC	97 (66.0)	19 (73.1)	
LUAD	28 (19.0)	5 (19.2)	
Others^b^	22 (15.0)	2 (7.7)	
dNLR, median (IQR)	-0.22 (-1.56–0.28)	-0.54 (-1.69–0.12)	0.481
dMLR, median (IQR)	0.24 (-0.05–0.92)	0.12 (-0.08–0.76)	0.253
dPLR, median (IQR)	-15.03 (-57.85–27.85)	-36.32 (-64.59–6.35)	0.088
dPAR, median (IQR)	-1.35 (-2.96–0.27)	-2.26 (-4.14–1.19)	0.123
dSII, median (IQR)	-185.81 (-530.95–46.37)	-344.67 (-633.26–236.87)	0.196
dSIRI, median (IQR)	-0.17 (-0.99–0.22)	-0.47 (-1.04–0.03)	0.279
dPNI, median (IQR)	-1.35 (-4.25–3.00)	-0.62 (-2.44–3.51)	0.930
dPIV, median (IQR)	-106.83 (-393.47–23.93)	-144.54 (-333.89–45.24)	0.221
MPR			0.491
Yes	101 (68.7)	20 (76.9)	
No	46 (31.3)	6 (23.1)	
pCR			0.09
Yes	58 (39.5)	15 (57.7)	
No	89 (60.5)	11 (42.3)	

IQR, interquartile range; LUSC, lung squamous carcinoma; LUAD, lung adenocarcinoma; LELC, lung lymphoepithelioma-like carcinoma; dNLR, dynamic change of neutrophil–lymphocyte ratio; dMLR, dynamic change of monocyte–lymphocyte ratio; dPLR, dynamic change of platelet–lymphocyte ratio; dPAR, dynamic change of platelet–albumin ratio; dSII, dynamic change of systemic immune inflammation index; dSIRI, dynamic change of system inflammation response index; dPNI, dynamic change of prognostic nutritional index; dPIV, dynamic change of pan-immune-inflammatory value; MPR, major pathological response.

a Diagnosed based on the AJCC criteria (8th edition).

b Including LELC, adenosquamous carcinoma, large cell neuroendocrine carcinoma.

Bold values mean statistical significance.

### 3.3 Identifying a MPR signature based on dPBI

Using NSCLC patients in the training cohort, we investigated the value of dPBI in predicting MPR for NSCLC patients receiving neoadjuvant immunochemotherapy (**Figure 1**). We initially performed the LASSO regression algorithm to integrate the longitudinal dynamics of above eight peripheral blood inflammatory indexes at the optimal value of -4.28 of log(e)λ with minimal bias, four dPBI without zero coefficients, i.e., dMLR, dPLR, dPAR, and dPNI, were screened out as indicators associated with MPR to further construct the MPR signature, which was named as the MPR score (**Supplementary Figures 1A, B**). The specific formula of MPR score was as follows: MPR score = dMLR * -3.70E-01 + dPLR * -8.81E-04 + dPAR * -7.65E-02 + dPNI * -6.61E-03.

### 3.4 Predictive value of the MPR score for MPR

Subsequently, we integrated the MPR score as well as clinical characteristics into the univariate and multivariate logistics analysis to further identify the predictive factors of MPR. As shown in **Table 2**, the univariate logistic analysis demonstrated that gender (*P* = 0.028), smoking history (*P* = 0.028), histology (LUSC *versus* LUAD, *P* = 0.001; LUSC *versus* others, *P* = 0.001), differentiation degree (*P* = 0.001), and the MPR score (*P* = 0.016) were statistically significant for MPR. Before multivariate logistics analysis, we tested the multicollinearity through variance inflation factor (VIF) and tolerance of MPR score, gender, histological type, smoking history, and differentiation degree. The VIF of score, gender, histological type, smoking history, and differentiation degree is 1.044, 1.606, 1.254, 1.762, and 1.050, respectively, which were less than 5. The tolerance of score, gender, histological type, smoking history, and differentiation degree is 0.958, 0.623, 0.798, 0.567, and 0.952, respectively, which were larger than 0.1.The multivariate logistics analysis further revealed that the MPR score (*P* = 0.028, OR 4.756; 95% CI 1.183–19.116), differentiated degree (*P* < 0.001, OR 4.432; 95% CI 2.015–9.750), and histological type (LUSC *versus* LUAD, *P* < 0.001, OR 0.139; 95% CI 0.046–0.415; LUSC *versus* others, *P* = 0.007, OR 0.182; 95% CI 0.053–0.621) were independent predictors of MPR for NSCLC patients who underwent neoadjuvant immunochemotherapy.

**Table 2:** Univariate and multivariate logistics analysis of MPR for the training cohort.

Factors	Univariate analysis		Multivariate analysis	
	OR (95% CI)	*P*-value	HR (95% CI)	*P*-value
Gender	0.306 (0.16–0.882)	**0.028**	0.713 (0.166–3.065)	0.650
Age (year)	1.030 (0.986–1.077)	0.186		
BMI	0.954 (0.857–1.061)	0.383		
Smoking history	2.373 (1.096–5.144)	**0.028**	2.313 (0.696–7.683)	0.171
cT stage^a^	1.009 (0.680–1.498)	0.963		
cN stage^a^	1.180 (0.797–1.749)	0.408		
Histology				
LUSC	Ref			
LUAD	0.211 (0.086–0.517)	**0.001**	0.139 (0.046–0.415)	**<0.001**
Others^b^	0.203 (0.076–0.540)	**0.001**	0.182 (0.053–0.621)	**0.007**
Differentiation degree				
Moderate	Ref		Ref	
Poor or undifferentiated	3.172 (1.599–6.296)	**0.001**	4.432 (2.015–9.750)	**<0.001**
Score	4.234 (1.301–13.777)	**0.016**	4.756 (1.183–19.116)	**0.028**

LUSC, lung squamous carcinoma; LUAD, lung adenocarcinoma; LELC, lung lymphoepithelioma-like carcinoma.

a Diagnosed based on the AJCC criteria (8th edition).

b Including LELC, adenosquamous carcinoma, large cell neuroendocrine carcinoma.

Bold values mean statistical significance.

### 3.5 Development of the predictive nomogram for MPR

**Figure 3:**
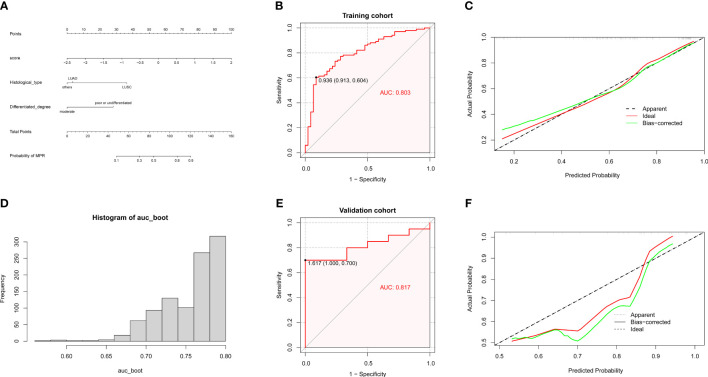
Development and validation of the nomogram. **(A)** Nomogram for predicting MPR for NSCLC patients after neoadjuvant immunotherapy, according to which each variable could be assigned a score on the point scale. By adding up the total points, we could determine the estimated probability of MPR. **(B)** Receiver operating characteristic curve of the nomogram in training cohort. **(C)** Calibration plots for the nomogram in the training cohort. Predicted and actual MPR probability were respectively plotted on the X-axis and the Y-axis. The 45° dashed lines through the coordinate origin represent the excellent calibration models. **(D)** Distribution of AUC for 1,000 times bootstrapping. **(E)** Receiver operating characteristic curve for the nomogram in the validation cohort. **(F)** Calibration plots for the nomogram in the validation cohort. The predicted and actual MPR probability were respectively plotted on the X-axis and the Y-axis. The 45° dashed lines through the coordinate origin represent the excellent calibration models.

Based on the aforementioned three independent predictors from the above-mentioned multivariate logistics analysis, i.e., the MPR score, differentiated degree, and histology, a predictive model to predict MPR derived from neoadjuvant immunochemotherapy was developed and visually presented as a nomogram (**Figure 3A**). Through this nomogram, the corresponding score of these three factors were determined by their projection onto the point scale. We subsequently summed up their total scores and projected it to the total point scale and then projected downward onto the bottommost line to predict the probability of MPR of NSCLC patients treated with neoadjuvant immunochemotherapy. It was evident that higher total scores in patients were associated with an increased probability of MPR. For easy use of our nomogram, we provided the point for each factor and the probability of MPR associated with the different total points (**Supplementary Table S2**).

### 3.6 Assessment of predictive performance of the nomogram for MPR

We draw ROC curves and calibration curves of this nomogram in the training cohort and the validation cohort to evaluate its predictive performance. In the training cohort, the AUC value of ROC was 0.803, which represented a high predictive efficacy in predicting MPR (**Figure 3B**). The calibration curve displayed a high agreement between the virtual (Y-axis) and predicted (X-axis) probabilities of MPR (**Figure 3C**), which indicated a satisfactory predictive performance in internal validation. In addition, we further performed internal bootstrap validation with 1,000 repetitions. The main AUC after 1,000 times of bootstrapping was 0.752 (IQR 0.682–0.795). The histogram in **Figure 3D** showed the distribution of AUC for 1,000 times of bootstrapping, and more than half of the repetitions of AUC were closed to 0.8.

Similarly, the predictive performance of this nomogram was validated in the validation cohort. This nomogram also showed a high predictive performance in the validation cohort with an AUC of 0.817 (**Figure 3E**). The calibration curve also displayed a relatively good agreement between the virtual and predicted probabilities of the MPR (**Figure 3F**). Moreover, DCA plots demonstrated that a net benefit for NSCLC patients could be obtained when patients utilize this nomogram in neoadjuvant immunochemotherapy (**Supplementary Figures S2A, B**).

### 3.7 Prognostic value of MPR and MPR score for EFS

**Figure 4:**
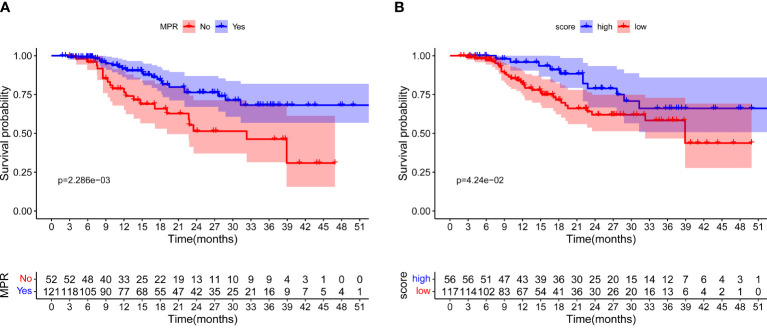
Event-free survival for all patients. **(A)** Survival curves for patients with MPR and non-MPR. **(B)** Survival curves for patients with high and low MPR score, respectively.

The median follow-up time of all 173 patients was 26.7 months, and 43 recurrence or death events were observed. At first, we attempted to explore the association between MPR and EFS outcomes. As shown in **Figure 4A**, significantly better EFS of NSCLC patients with MPR after neoadjuvant immunochemotherapy than those without MPR was seen in the Kaplan–Meier curves (log-rank test *P* = 0.002). Then, maximally selected log-rank statistics determined 0.23 as the cutoff value of MPR score, and 56 of 173 patients had a high MPR score (**Supplementary Figure S3**). As shown in **Figure 4B**, an apparently longer EFS time was observed in patients with high MPR score than those with low MPR score (log-rank test *P* = 0.042). We also explored the prognostic value of gender, BMI, smoking history, histological type, differentiated degree, and cT and cN stage. Only pCR showed statistical significance (*P* = 0.001) in univariable Cox regression analysis (**Supplementary Figure S4**).

### 3.8 MPR signature and dynamic changes of exosomal miRNA profile

**Figure 5:**
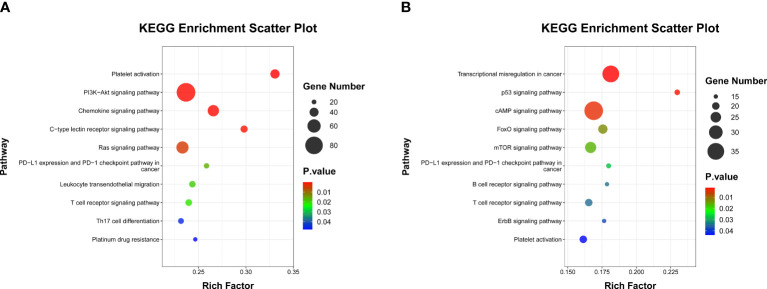
Scatter plot of KEGG enrichment analysis. **(A)** Pathways involved in immune regulation and signal transduction of three miRNAs which positively correlated with MPR signature. **(B)** Pathways involved in immune regulation and signal transduction in two miRNAs which negatively correlated with MPR signature.

As inflammatory status can suppress innate and adaptive immune responses (31), we next sought to explore whether this peripheral MPR signature was related to distinct transcriptional signatures in the context of neoadjuvant immunochemotherapy. We focused on paired analyses of baseline and post-ICI plasma of 12 patients with NSCLC in the training cohort, from which we firstly purified the exosomes through the GS Reagent DF Kit, and by means of transmission electron microscopy, we observed the small double-leaflet membrane particles (**Supplementary Figure S5**). Then, we performed miRNA sequencing from plasma exosomes, and a differential analysis found 549 DE miRNA between baseline and post-ICI samples. Compared with baseline samples, 293 miRNAs were upregulated, and 256 miRNAs were downregulated in post-ICI samples (**Supplementary Table S3**). After excluding miRNAs with low expression, we further calculated the longitudinal dynamic change of the expression of DE miRNAs and performed Pearson correlation analysis with the predictive score of our MPR score. As shown in **Table 3**, five miRNAs were significantly related to our MPR score, among which two miRNAs were negatively associated with the MPR score and three miRNAs showed a positive correlation with the MPR score. Subsequently, we predicted the targeted genes of these five miRNAs and performed KEGG enrichment analysis to explore the potential function of these miRNAs. These analyses revealed enrichments in some immune regulation and signal transduction pathways, specifically speaking, miRNAs positively correlated with the MPR score showed enrichments in platelet activation, PI3K-Akt signaling pathway, chemokine signaling pathway, PD-L1 expression and PD-1 checkpoint pathway, leukocyte transendothelial migration, Th17 cell differentiation, and so on. Other two miRNAs negatively correlated with the MPR score were enriched in transcriptional misregulation in cancer, p53 signaling pathway, mTOR signaling pathway, B cell receptor signaling pathway, T cell receptor signaling pathway, and so on (**Figures 5A, B**).

**Table 3:** miRNA significantly correlated with MPR signature.

miRNA	*R* ^2^	*P*-value	Relation
hsa-miR-5586–5p	-0.66341	0.019	Negative
hsa-miR-3159	-0.66068	0.019	Negative
hsa-miR-6124-p5	0.681547	0.014	Positive
hsa-miR-501–5p	0.782831	0.002	Positive
hsa-miR-3614–5p	0.628972	0.028	Positive

## 4 Discussion

Mechanically speaking, an intact host immunity status tends to release or expose to tumor neoantigens to activate tumor-specific T cells; thus, preoperative immunotherapy has much advantage to eradicate tumor cells and micrometastases (32). ICI-based neoadjuvant therapy has been recommended as a prior antitumor strategy for patients with locally advanced NSCLC in consideration of its remarkable tumor-killing effects and sustaining clinical benefits (2–4). Due to the heterogeneous response rate to neoadjuvant immunochemotherapy in NSCLC patients, an accurate and reproducible prediction of antitumor immune responses among patients with NSCLC who underwent ICI-based neoadjuvant regimens is much essential to optimize patient benefits, improve clinical outcomes, and reduce social medical cost. In this retrospective study, we monitored the longitudinal dynamic changes in peripheral blood inflammatory indexes in NSCLC patients treated with preoperative immunochemotherapy. Utilizing the LASSO algorithm, we revealed four dPBI (dMLR, dPLR, dPAR, and dPNI) to be associated with MPR and further developed a MPR score to model their longitudinal dynamics. The MPR score was subsequently identified as an independent predictor of MPR; a predictive model on the basis of this MPR score was subsequently constructed and showed good stratification performance on MPR prediction in NSCLC patients treated with neoadjuvant immunochemotherapy. Besides that, miRNA transcriptomic analysis of plasma exosomes represents a significant correlation between the MPR score and immune cell regulations as well as immune-related pathways. Taken together, our study suggested that dPBI might be an indirect reflection of antitumor immune responses derived from neoadjuvant immunochemo-therapy and, ultimately, favorable clinical outcomes.

The value of inflammation-nutrition-related biomarkers in predicting the prognosis and immunotherapeutic efficacy in patients with NSCLC has become a research hotspot in recent years (13, 33–36). Diem et al. found that baseline NLR ≥5 was independently correlated with the inferior overall survival (OS) of nivolumab-treated patients with advanced NSCLC (33). Similarly, Sun et al. reported that the high baseline NLR was an independent predictor of poor pathological response and shorter disease-free survival for resectable NSCLC patients receiving neoadjuvant chemotherapy plus ICIs (34). Sekine et al. revealed that a rapid decrease of the peripheral MLR was significantly associated with the efficacy of nivolumab monotherapy in advanced NSCLC (35). Advanced NSCLC patients with higher baseline PNI exhibited better clinical outcomes from immunotherapy (36). On the one hand, inflammation, as the recognized hallmark of tumors, reflects the overall immune function of the body and is substantially associated with anti-tumor immunity. Previous researches have reported that the expression pattern of inflammation-related genes, proteins, and cytokines was a vital part of the TME (37, 38). On the other hand, the systemic inflammatory-nutrient status plays a vital role in tumor progression and patients’ survival. Usually, cancer patients tend to experience malnutrition due to vigorous metabolism and abnormal proliferation of tumor cells, leading to loss of muscle, fat, and body weight. Furthermore, malnutrition might damage the immune system, causing an imbalance between immune-suppression and tumor proliferation; hence, the body’s immune system fails to eliminate tumor cells, and the possibility of cancer-related death finally increases (39). Thus, considerable efforts on combining peripheral inflammatory and nutrition-related indexes to predict the prognosis and immunotherapeutic efficacy of NSCLC patients have been conducted (6, 13–16).

Although the underlying causal effects of the above-mentioned association are still unclear, several hypotheses can be proposed. Circulating classical monocytes extravasate into tissues and further differentiate into macrophages. Tumor-associated macrophages (TAMs) contribute to tumor progression in diverse ways, including promoting genetic instability, stimulating angiogenesis and lymphangiogenesis, facilitating tumor cell extravasation, survival, proliferation, and persistent growth, nurturing tumor stem cells, promoting epithelial–mesenchymal transition, remodeling the extracellular matrix, priming the premetastatic site, and supporting metastasis. TAMs also induce immunosuppression through secreting cytokines such as IL-10 and TGF-β to prevent tumor cells from being attacked by natural killer and T cells during progression and after recovery from chemotherapy or anti-tumor immune (40–42). Low grade TAMs correlate with better prognosis and improve overall survival (41). Platelets have been shown to actively contribute to the process of tumor metastasis, and these bind to circulating tumor cells (CTCs), forming a platelet shield around CTCs, which protect CTCs from shear stress by reducing the exerted force, avoid CTCs from being recognized by the immune system to facilitate immune evasion, and provide adhesive sites on the wall of blood vessels to promote tumor cells to extravasate into tissues (43, 44). Depleted platelet has been observed to be correlated with decrease in tumor growth (45). In contrast, peripheral albumin level sensitively reflects the nutrition status of the body; a low albumin causes a high level of malnutrition and is related to poor clinical outcomes for lung cancer patients (46). Lymphocytes, especially CD8+ T cells, play an important component in anti-tumor immune response through inhibiting tumor cell proliferation and migration and inducing cytotoxic deaths. CD8+ T cells not only directly kill cancer cells via perforin and granzyme pathways or the Fas/Fas ligand pathway but also indirectly destroy tumors through secreting cytokines such as IFN-γ and TNF-α (14, 47, 48). High peripheral blood lymphocytes indicate stronger endogenous anti-tumor capacity in the body, and lymphocytopenia is associated with poor survival in numerous settings as tumors might induce lymphocyte apoptosis both within the TME and in peripheral circulation as a means of avoiding immune recognition (49). In line with this notion, we focused on the dPBI and identified a MPR signature, named MPR score, based on these dynamics during neoadjuvant immunochemotherapy. Furthermore, we revealed a significant correlation between an increased MPR score during the treatment, that is, reduction of MLR, PLR, PAR, and PNI, with a higher possibility of MPR. Additionally, NSCLC patients with MPR after ICI-based regimens of neoadjuvant treatment presented significantly better clinical responses (EFS) than those without MPR.

Previous homogeneous research usually paid attention to the value of pretreatment peripheral inflammatory biomarkers in predicting the anti-tumor immune responses in NSCLC patients (13), which neglected the dynamic nature and plasticity of the tumor–immune system interplay of the immune checkpoint blockade and was not very robust. Analyses integrated with tumor-intrinsic and immune cell-focused features showed that nuanced characteristics of the tumor genomic landscape together with proinflammatory signatures in TME could better distinguish responding from non-responding tumors (9, 50, 51)—for instance, the recruitment of TAMs to tumors is mainly mediated by a range of tumor-derived chemokines, including CCL2, VEGF, CCL5, and CSF1 (41). Ali HR et al. identified that immunotherapy distinctively remodeled the tumor structure; non-responders were characterized by increasing levels of CD15+ cells (a carbohydrate blood group antigen expressed by neutrophils and monocytes), while key leukocytes, such as T cells, increased dramatically on treatment and the dynamics of macrophages and dendritic cells mirrored that of T cells (17). In addition, a significant overlap between responders and non-responders to ICIs exists for biomarkers tested just based on the analysis of pretreatment tumor biopsies (52, 53). Wargo et al. obtained longitudinal tumor biopsies of metastatic melanoma patients treated with ICIs. Immune profiling analysis of immune cell infiltrates in TME showed that there was no difference in any of the measured markers between responders *versus* non-responders to CTLA-4 blockade at the pretreatment time point, while an analysis of early on-treatment tumor biopsies revealed a significantly higher density of CD8+ T cells in responders than non-responders to CTLA-4 blockade. Although further immune profiling analysis for patients treated with anti-PD-1therapy had a modestly statistically significant difference in the density of T cells subsets in the pretreatment baseline samples of responders compared to non-responders, their values were largely overlapping. In contrast, a profound and highly statistically significant difference in the expression of markers for T cell subsets and immunomodulatory molecules was shown in responders *versus* non-responders to therapy in early on-treatment tumor samples, with little to no overlap between groups (50). Therefore, our study provided novel evidence that assessing neoadjuvant immune-chemotherapy responses could be precisely achieved through minoring the dPBI during the treatment, rather than solely according to examination at baseline. Theoretically, ICIs and chemotherapy can influence the proliferation, migration, chemotaxis, and activation of peripheral blood mononuclear cell (PBMC) to killing tumor cells, and we guess tumor cells counteract the killing through a series of biological reaction such as secreting exosomes containing characteristic substances, and this process can show up in the dPBIs (54–57). Therefore, we performed exosomal miRNA seq analysis to dissect the possible intrinsic relationship between the MPR score and MPR. Furthermore, the exosomal miRNA sequencing and DE analysis obtained at baseline and posttreatment time points showed a significant Pearson correlation among the certain miRNAs and our MPR score. The targeted genes of the abovementioned miRNAs as well as their enriched pathways dissected the possible intrinsic relationship between the MPR score and MPR, that is, patients with a high MPR score might not have a weakened anti-tumor immune, such as decreased platelet activation. Similar to a previous study, we report that early on-treatment tumor samples of patients treated with immunotherapy showed significant DEGs in responders and upregulated DEGs related to processes such as antigen presentation, T cell activation, and T cell homing, but there were no significant differences in targeted gene expression profiling at pre-treatment ICIs (50). Hence, our MPR score might represent another strategy through which clinicians could best predict responders to neoadjuvant immunochemotherapy.

Despite our MPR score integrating dPBI during neoadjuvant immunochemotherapy and the considerable predictive efficiency of this MPR score-based model, several limitations should be acknowledged for this study. First, confounding factors or uncaptured sources of bias should be noted due to the retrospective nature of this investigation, and the relatively small sample size of the validation cohort was limited. Although we adopted an independently managed cohort (NCT04304248) to evaluate the predictive capacity of the MPR model, larger-scale studies in a prospective design and external validation are warranted in the future. Second, although we dissected the correlation between the MPR score and miRNA transcriptomic analysis of plasma exosomes, we did not explore the intratumoral T cell clonal dynamics in peripheral blood during this ICI-based neoadjuvant treatment; hence, if this MPR score could be linked with the expansions in peripheral effector lymphocytes are worthy of consideration. However, our findings are strengthened by the consistency with another similar study in NSCLC patients, which demonstrated that peripheral inflammatory indexes captured the T cell repertoire reshaping post-ICI. Third, though this study revealed the prognostic value of MPR, pCR is the most important thing in the treatment strategy. In the future, prospective exploration of markers of pCR will be a more valuable work.

## 5 Conclusion

We comprehensively analyzed the dPBI and constructed the MPR score for non-invasive prediction of neoadjuvant immunochemotherapy responders for patients with NSCLC. Furthermore, our analysis supported the notion that this MPR score was associated with underlying transcriptome dynamics in plasma exosomes in the quality of the antitumor immune response in the TME, providing the potentially biological foundation to dissect their association. Hence, for patients with NSCLC planned for neoadjuvant immunochemotherapy, integrative predictive models of response incorporating this non-invasive, readily available biomarkers might help to identify patients who are less likely to obtain clinical outcome benefits on ICI-based treatment, allowing for rapid adaptive changes in therapeutic strategy.

## Data availability statement

The datasets presented in this study can be found in online repositories. The names of the repository/repositories and accession number(s) can be found below: PRJNA1127207 (SRA).

## Ethics statement

The studies involving humans were approved by the Institutional Review Board of Sun Yat-sen University Cancer Center. The studies were conducted in accordance with the local legislation and institutional requirements. The ethics committee/institutional review board waived the requirement of written informed consent for participation from the participants or the participants’ legal guardians/next of kin because this was a retrospective study.

## Author contributions

WZ: Formal analysis, Methodology, Writing – original draft, Writing – review & editing. CZ: Methodology, Writing – original draft, Writing – review & editing. FD: Formal analysis, Writing – original draft, Writing – review & editing. JX: Data curation, Investigation, Writing – review & editing. SD: Data curation, Investigation, Writing – review & editing. YL: Data curation, Investigation, Writing – review & editing. QY: Data curation, Investigation, Writing – review & editing. BR: Data curation, Investigation, Writing – review & editing. LL: Data curation, Investigation, Writing – review & editing. YZ: Data curation, Investigation, Writing – review & editing. ZZ: Conceptualization, Resources, Supervision, Writing – review & editing. HL: Conceptualization, Supervision, Writing – review & editing. JW: Conceptualization, Funding acquisition, Project administration, Supervision, Writing – review & editing.

## Supplementary material

Supplementary Figure 1 Identification of predictor of major pathological response among dynamics of peripheral blood inflammatory indexes (dPBI) in training cohort. **(A)** Selection of the optimal candidate dPBI in the LASSO model. **(B)** LASSO coefficients of dPBIs, each curve represents a dPBI.

Supplementary Figure 2 The decision curve analysis of the clinical value for the nomogram. **(A)** The decision curve of training cohort; **(B)** The decision curve of validation cohort.

Supplementary Figure 3 Determination of cut-off value of MPR score by the maximally selected log-rank statistics.

Supplementary Figure 4 The forest plot of univariable cox regression analysis of gender, BMI, smoking history, histological type, differentiated degree, cT and cN stage.

Supplementary Figure 5 Representative electron microscope image of purified plasma-derived exosome.
