# Supplementary figures and images for: Dynamics of peripheral blood inflammatory index predict tumor pathological response and survival among patients with locally advanced non-small cell lung cancer who underwent neoadjuvant immunochemotherapy: a multi-cohort retrospective study
Source: Front Immunol. 2024 Jul 23;15:1422717. doi: 10.3389/fimmu.2024.1422717 (PMC11300317; doi:10.3389/fimmu.2024.1422717)

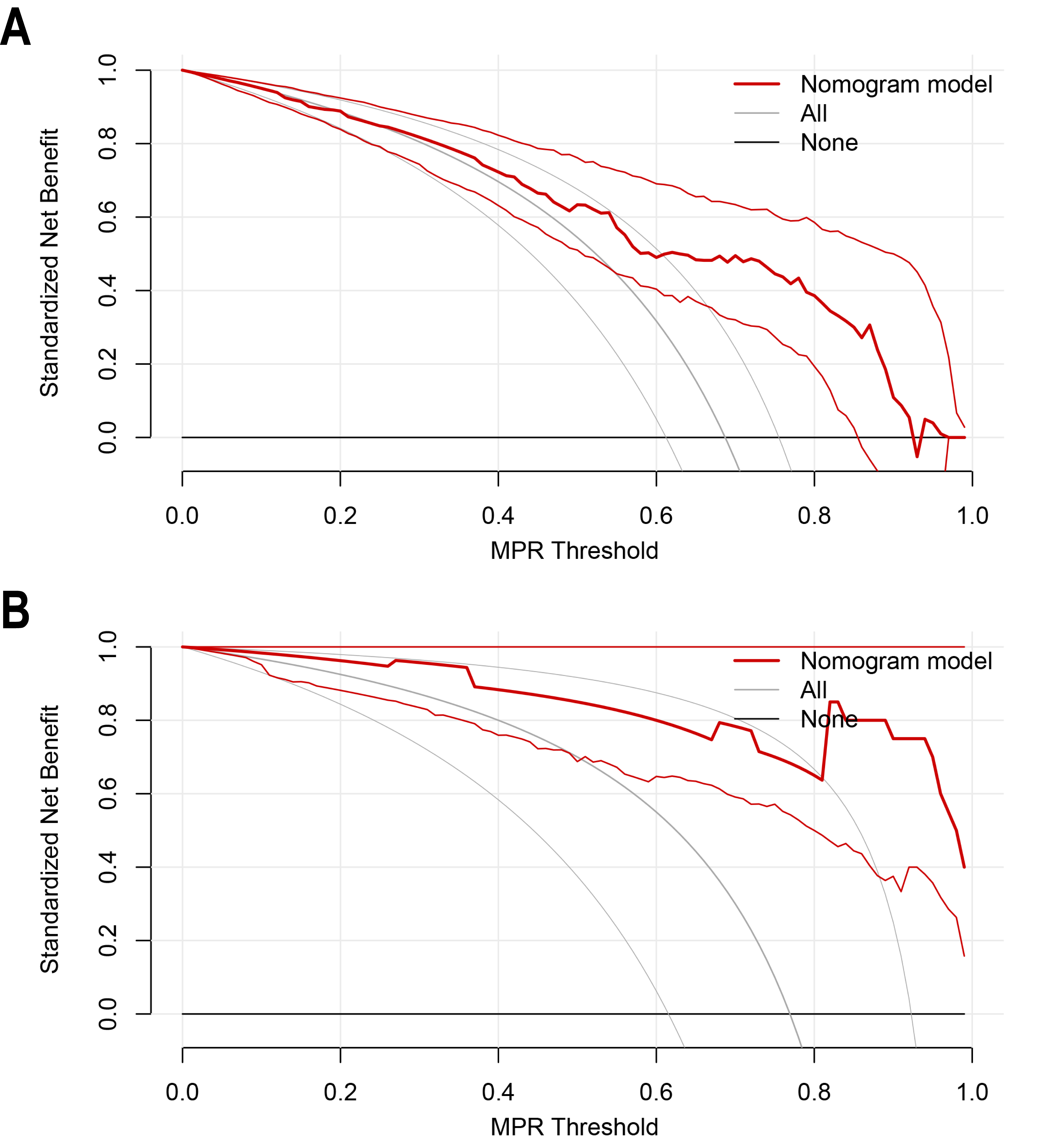

Supplement: Supplementary Figure 1 — Identification of predictor of major pathological response among dynamics of peripheral blood inflammatory indexes (dPBI) in training cohort. (A) Selection of the optimal candidate dPBI in the LASSO model. (B) LASSO coefficients of dPBIs, each curve represents a dPBI. [file Image_1.tif]

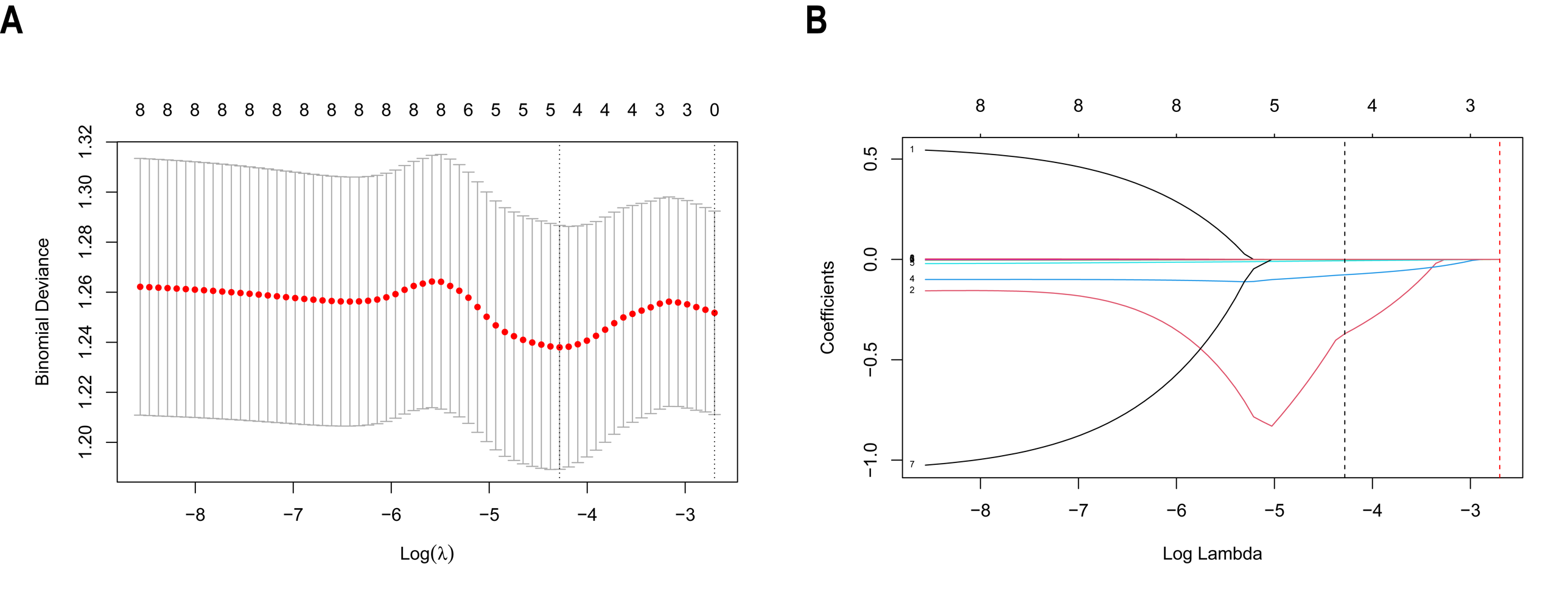

Supplement: Supplementary Figure 2 — The decision curve analysis of the clinical value for the nomogram. (A) The decision curve of training cohort; (B) The decision curve of validation cohort. [file Image_2.tif]

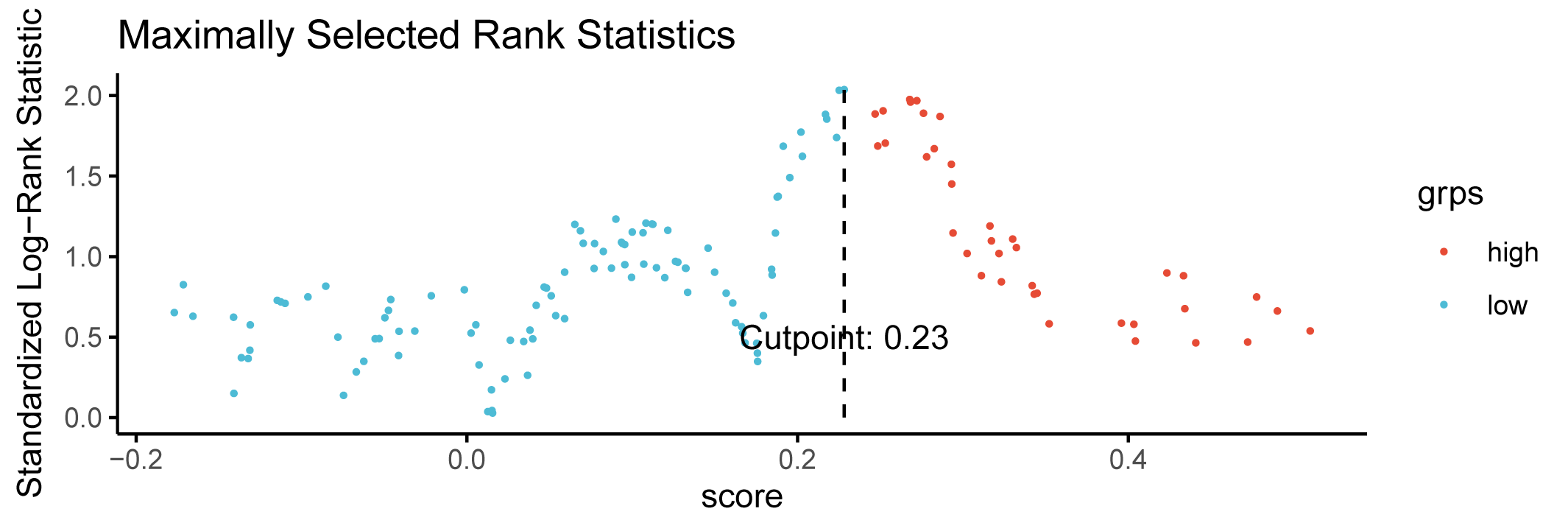

Supplement: Supplementary Figure 3 — Determination of cut-off value of MPR score by the maximally selected log-rank statistics. [file Image_3.tif]

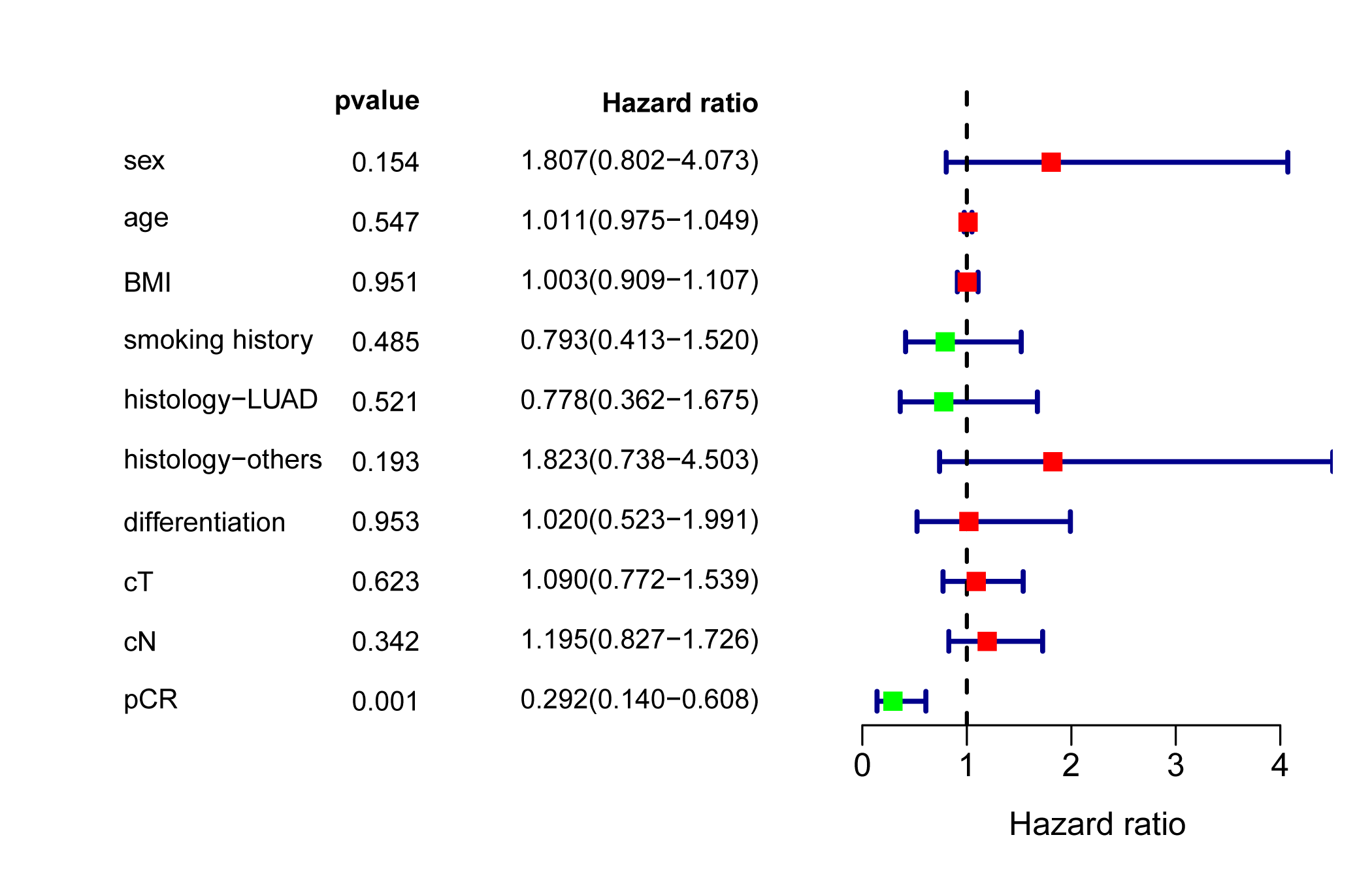

Supplement: Supplementary Figure 4 — The forest plot of univariable cox regression analysis of gender, BMI, smoking history, histological type, differentiated degree, cT and cN stage. [file Image_4.tif]

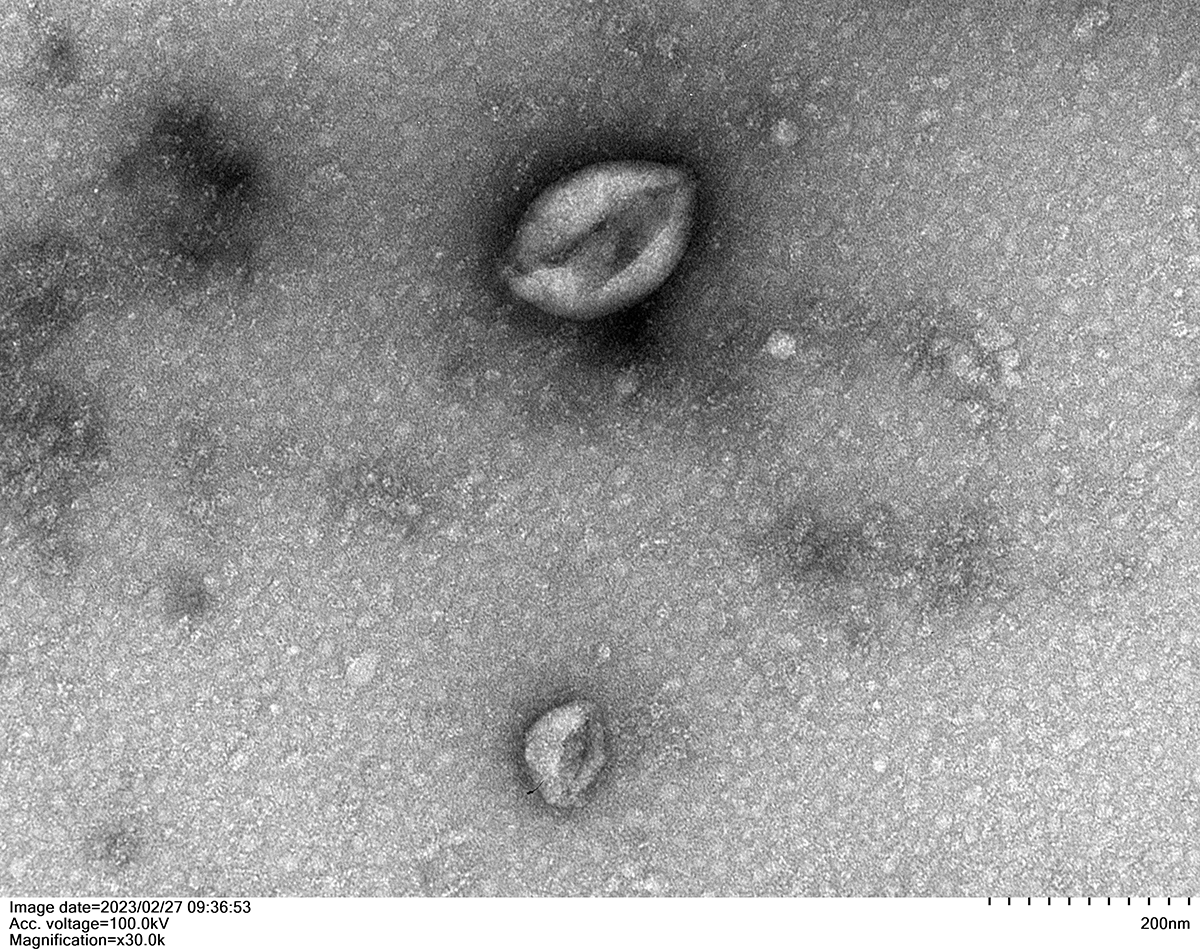

Supplement: Supplementary Figure 5 — Representative electron microscope image of purified plasma-derived exosome. [file Image_5.tif]
